# Evaluation of Intensity of Sleep Bruxism in Arterial Hypertension

**DOI:** 10.3390/jcm7100327

**Published:** 2018-10-05

**Authors:** Helena Martynowicz, Pawel Dymczyk, Marzena Dominiak, Klaudia Kazubowska, Robert Skomro, Rafal Poreba, Paweł Gac, Anna Wojakowska, Grzegorz Mazur, Mieszko Wieckiewicz

**Affiliations:** 1 Department of Internal Medicine, Occupational Diseases, Hypertension and Clinical Oncology, Wroclaw Medical University, 213 Borowska St., 50-556 Wroclaw, Poland; helenamar@poczta.onet.pl (H.M.); dymczykdentysta@gmail.com (P.D.); sogood@poczta.onet.pl (R.P.); ania.wojakowska@wp.pl (A.W.); grzegorzmaz@yahoo.com (G.M.); 2 Department of Oral Surgery, Wroclaw Medical University, 26 Krakowska St., 50-425 Wroclaw, Poland; marzenadominiak39@gmail.com (M.D.); kkazubowska@gmail.com (K.K.); 3 Division of Respiratory, Critical Care and Sleep Medicine, University of Saskatchewan, 107 Wiggins Road, SK S7N 5E5 Saskatoon, Saskatchewan, Canada; r.skomro@usask.ca (R.S.); 4 Department of Hygiene, Wroclaw Medical University, 7 Mikulicza-Radeckiego St., 50-345 Wroclaw, Poland; pawelgac@interia.pl (P.G.); 5 Department of Experimental Dentistry, Wroclaw Medical University, 26 Krakowska St., 50-425 Wroclaw, Poland; m.wieckiewicz@onet.pl (M.W.)

**Keywords:** sleep bruxism, arterial hypertension, obstructive sleep apnea

## Abstract

Sleep bruxism (SB) is a masticatory muscle activity during sleep that is characterized as rhythmic (phasic) or non-rhythmic (tonic). The recent hypothesis on the etiology of SB supports the role of the central and autonomic nervous systems. Therefore, in this study, we aimed to assess the intensity of SB in patients with arterial hypertension. A total of 70 adults participated in this study: 35 patients with hypertension (study group) and 35 normotensive subjects (control group). Data were recorded using home portable cardiorespiratory polygraphy. The bruxism episode index (BEI) in the study group was found to be significantly higher compared to the control group (3.4 ± 3.25 vs. 2.35 ± 2.29, *p* = 0.04). Hypertension, higher body mass index (BMI), lower values of mean oxygen saturation (SpO_2_), and a higher percentage of SpO_2_ < 90% constituted independent risk factors for increased BEI. These results suggest the need for special oral care in hypertensive patients, patients with higher BMI, lower values of SpO_2_ and a higher percentage of SpO_2_ < 90%.

## 1. Introduction

The American Academy of Sleep Medicine (AASM) defines bruxism as repetitive jaw-muscle activity characterized by clenching or grinding of the teeth and/or by bracing or thrusting of the mandible. According to International Classification of Sleep Disorders (ICDS-3) the clinical criteria for the classification of sleep bruxism (SB) include the following: (A) the presence of regular or frequent tooth-grinding sounds occurring during sleep; (B) the presence of one or more of the following clinical signs: (1) abnormal tooth wear consistent with the above reports of tooth grinding during sleep; (2) transient morning jaw muscle pain or fatigue, temporal headache, and/or jaw locking upon awakening consistent with the above reports of tooth grinding during sleep. Sleep-related movement disorders include restless legs syndrome, periodic limb movement during sleep, leg cramps, benign sleep myoclonus in infancy, propriospinal myoclonus at sleep onset, and rhythmic movement disorder [1].

With international consensus, sleep bruxism was defined as masticatory muscle activity during sleep that is characterized as rhythmic (phasic) or non-rhythmic (tonic) and is not a movement disorder or a sleep disorder in otherwise healthy individuals [2]. In the past, occlusal imbalance was considered as the primary etiological factor for bruxism; thus, dentists used to indicate occlusal adjustment [3] or occlusal stabilization splints [4]. However, SB is no longer considered to be related to occlusal discrepancies or is not considered to be a result of stress, anxiety, or depression [5,6,7]. The most recent hypotheses on the etiology of SB support the roles of the central and autonomic nervous systems in the genesis of masticatory muscle activity during sleep [8]. Most SB episodes occur in the light stages of sleep in association with sleep arousals [9], which are cortical brain activations lasting 3–15 s, with an accompanying increase in the heart rate and motor activity [10,11]. Smoking, the use of certain medications, and breathing problems can be considered as risk factors for SB [12]. Obstructive sleep apnea (OSA) syndrome is considered a risk factor for SB [13,14]; however, several studies have failed to confirm this correlation [15,16]. A polysomnographic study has confirmed the link between bruxism and insomnia, but there are no studies regarding the association between SB and stress and depression [17].

SB and periodic limb movement during sleep (PLMS) are classified as sleep-related movement disorders by the AASM. Both PLMS and cortical arousal are associated with an increase in blood pressure (BP), and PLMS has been shown to exhibit a higher surge in BP when co-occurring with sleep arousals [18,19]. Studies regarding the association between hypertension and bruxism are limited. Hypertension is the most common chronic cardiovascular disease in adults [20,21]. OSA is a major modifiable risk factor of hypertension [22]. A number of epidemiologic studies have shown an association between OSA and hypertension [23,24], particularly when nocturnal hypertension is concerned [25]. OSA is considered to be a possible risk factor for SB [26], but there are no studies that discuss whether hypertension could influence the prevalence or severity of SB.

Therefore, in this study, we aimed to assess the intensity of SB in patients with arterial hypertension (HTN) compared to non-hypertensive individuals.

## 2. Material and Methods

In this study, 70 adults participated: 35 patients with hypertension (study group) and 35 normotensive subjects (control group). The enrolled 35 patients with hypertension had a history of HTN and were taking anti-hypertensive medications. The control group subjects, who had no history of HTN, did not take any anti-hypertensive medication. The patients were enrolled between March 2017 and April 2018 in two dental clinics located in Wroclaw, Poland by qualified dentists. Inclusion criteria were as follows: age between 18 and 90 years, presence of HTN in anamnesis, suspicion of SB and/or OSA based on self-report and clinical inspection, and that patients were willing to participate in this study. Exclusion criteria were as follows: avoidance of performance of the home sleep study, presence of neurological disorders and/or neuropathic pain, respiratory insufficiency, patients being treated with or addicted to analgesic drugs and/or drugs that affect muscle and breath function, presence of malignancy and severe mental disorders, active inflammation, and cognitive disability.

The Epworth Sleepiness Scale (ESS) was used to measure the subjects’ level of daytime sleepiness. This scale consists of eight items that measure a subject’s habitual likelihood of dozing or falling asleep in common situations. The ESS total score (0–24) represents the sum of the scores for the individual items. Scores above 10 were considered abnormal [27]. In addition to ESS, the questionnaire collected data regarding symptoms and comorbidities of OSA and smoking status. Patients also completed the STOP (Snoring; Tiredness/sleepiness/fatigue; Observed apnea; blood Pressure) questions and answered four yes/no questions (Bang self-reported) about their BMI (weight and height), age, neck circumference, and gender. The body mass index (BMI, calculated as weight in kilogram divided by square of height in meter) was calculated. Demographic characteristics are presented in Table 1.

Data were collected using home portable cardiorespiratory polygraphy (Nox-T3 Portable Sleep Monitor, Nox Medical, Reykjavík, Iceland). This device, used to confirm SB and OSA diagnosis, has been previously described [28]. Nox T3 is a Level 3 portable monitor, which registers the bilateral masseter electromyography (EMG) and the audio evaluation (the sounds of bruxism and snoring were recorded with a microphone integrated within the device). A minimum of two audible tooth-grinding episodes coincident with the EMG bursts confirmed the diagnosis of bruxism. Bruxism episodes were scored according to the standards of the AASM in three forms: phasic, tonic, and mixed. To be considered as SB, EMG activity had to be at least twice the amplitude of the background EMG. EMG bursts should not be separated by more than 3 s to be considered part of the same episode [29]. The number of total episodes per hour was calculated and used as the main outcome variable (BEI—bruxism episode index). Moreover, the number of phasic, tonic, and mixed episodes per hour was computed separately. Episodes could occur spontaneously or in conjunction with respiratory events. The following respiratory signals were noted during the portable monitor recordings: nasal pressure, rib cage and abdominal movement by inductance plethysmography, snoring, body position, activity, as well as heart rate and arterial oxygen saturation (SpO_2_) with finger pulse oximetry. Respiratory events were recorded and assessed in accordance with the standards of the AASM. Abnormal respiratory events were scored from the pressure airflow signal and evaluated according to the standard criteria of the AASM Task Force [30]. Apneas were defined as the absence of airflow for ≥10 s. Hypopnea was defined as a reduction in the amplitude of breathing by ≥30% for ≥10 s with a ≥3% decline in blood oxygen saturation.

The scoring and manual analysis of clinically collected data was performed by a qualified physician (H.M.) from the Sleep Laboratory at the Wroclaw Medical University, Poland.

Statistical analysis was conducted using the “Dell Statistica 13” software (Dell Inc., Aliso Viejo, CA, USA). Quantitative data are presented as mean and standard deviation. Qualitative variables are expressed as a percentage. Significant statistical differences between arithmetic means were determined by the Mann–Whitney *U* test and the between-group percentage by the chi-squared test. In order to determine the relationship between the tested variables, we conducted correlation as well as univariate and multivariate regression analysis. The statistical significance was set at a *p*-value < 0.05.

This study was approved by the Local Ethical Committee (ID KB-195/2017). Patients signed a consent form for participating in this study.

## 3. Results

The mean age of all participants was 57.34 ± 7.77 years. Diabetes and ischemic heart disease were diagnosed in 10.85% (*n* = 7) and 7.14% (*n* = 5) of the whole study population (*n* = 70), respectively. The mean BMI was found to be 27.39 ± 4.83 kg/m^2^. Smokers constituted 12.69% of all participants. The prevalence of OSA was found to be 55.29% (*n* = 38) in patients with hypertension and 21.43% (*n* = 15) in control group.

The mean age was similar in both groups (57.74 ± 7.92 years in the study group vs. 56.94 ± 7.71 years in the control group, respectively). There were no statistically significant differences in sleepiness scores in both groups. Study group demonstrated a higher mean score on the STOP-BANG scale than that of the control group (4.94 ± 1.72 vs. 3.14 ± 1.83, *p* = 0.001). (Table 1). The prevalence of coronary artery disease (CAD), stroke, and myocardial infarction were similar in both groups. The presence of two cases of patients after stroke (6.1%) and myocardial infarction (6.1%) in the normotensive group was accidental and did not affect the results of the study.

The prevalence of SB in both groups was found to be similar. In the study group, SB was diagnosed in 51% of them (*n* = 18) as compared to 49% in the control group (*n* = 17). In the study group, severe bruxism (BEI > 4) was diagnosed in 43% (*n* = 15) of them as compared to 23% (*n* = 8) in the control group. BEI in the study group was significantly increased compared to the control group (3.4 ± 3.25 vs. 2.3 ± 2.29, *p* = 0.04) (Table 2). Phasic bruxism was found to be increased in the study group as compared to the control group (1.09 ± 1.42 vs. 0.54 ± 0.76, *p* = 0.04). The mean values of tonic and mixed bruxism were found to be similar in both the groups.

The apnea–hypopnea index (AHI) in the study group was found to be 19.30 ± 17.59, which was found to be statistically increased compared to the control group (12.39 ± 10.86, *p* = 0.04).

In the complete study group, possible independent risk factors for BEI (as the dependent variable) were found on the basis of univariate linear regressions. In the analysis, variables possibly confounding the results were considered: anthropometric data (age, BMI, and gender), cardiovascular diseases (arterial hypertension, diabetes, CAD, myocardial infarction, and stroke), frequency of smoking habit and respiratory parameters (AHI, ODI, snoring, OA, MA, CA, hypopnea, Cheyne–Stokes, mean SpO_2_, min SatO_2_, SpO_2_ < 90%, mean oxygen desaturation, mean heart rate, maximum heart rate, and minimum heart rate). Next, with the use of a multivariate stepwise regression analysis of statistically significant variables from univariate linear regressions, a final model was obtained with the BEI as the dependent variable. The model—BEI = 57.22 + 2.05 HT + 0.12 BMI − 0.62 mean SpO_2_ + 0.05 SpO_2_ < 90% ± 1.02—was characterized by the higher value of the determination coefficient (*R*^2^ = 83.17%) and the lower *p*-value (*p* = 0.009). Based on the regression model that was obtained, it was shown that hypertension, higher BMI, lower values of mean SpO_2_, and a higher percentage of SpO_2_ < 90% constitute independent risk factors for the increased BEI (as the dependent variable; Table 3). The covariance was not assessed because of the relatively small sample size.

## 4. Discussion

The most interesting result of this study is the higher mean BEI in the study group as compared to the control group. The probable explanation for this phenomenon is the increased sympathetic activity in hypertension and SB; however, in this study, we did not investigate the role of sympathetic activity in SB. It is worth noting that an increased spillover of catecholamines from the kidney and heart to the plasma is intimately linked with the development and progression of hypertension [31]. Catecholamines may regulate orofacial movements through the premotor brainstem nuclei, which are related to masticatory control, as well as through forebrain areas related to autonomic and stress responses [32]. Thus, increased levels of catecholamines in hypertension may promote or intensify orofacial movements. Individuals with SB have higher levels of urinary catecholamines [33], which is in agreement with this hypothesis. Interestingly, clonidine—a centrally acting agonist to the presynaptic alpha2A receptor—has been used for decades in the treatment of hypertension. Moreover, it was shown that it may decrease sleep bruxism frequency by 61% [34]. It has been reported that clonidine decreases circulating catecholamines, thereby attenuating sympathetic activity [35,36], which supports our results and hypothesis.

The primary question with respect to the association of bruxism and hypertension is which of them is the primary cause and which of them is the consequence. Nashed et al. showed that SB is associated with the fluctuations in BP during sleep, and an increase in BP was found to be significant for RMMA/SB (rhythmic masticatory muscle activity) associated with cortical arousals, body movements, and cortical arousal with body movement [37]. Thus, it is possible that episodes of bruxism lead to a blood pressure surge; however, on the other hand, persistent hypertension may promote or intensify bruxism episodes. However, we have demonstrated that not only hypertension, but also higher BMI, lower values of mean SpO_2_, and higher percentages of SpO_2_ < 90% constitute independent risk factors for increased BEI. Numerous risk factors for SB in the general population have been described. OBS, snoring, sleepiness, alcohol, caffeine, smoking, stress and anxiety are well-known risk factors for SB [38]. Newly identified factors associated with bruxism predominantly concern diseases such as reflux esophagitis, depression, or nocturnal frontal lobe epilepsy [26]. Data concerning an association between BMI and bruxism are very limited; however, increased BMI is associated with sympathetic activity and increased plasma catecholamine levels as in hypertension [39]. Moreover, decreased salivary flow was reported both in obesity and bruxism [40,41]. Decreased mean SpO_2_ and a higher percentage of SpO_2_ < 90% commonly occurs in OBS, which is considered a risk factor for bruxism. Moreover, it was previously shown that transient hypoxia is potentially associated with the onset of episodes of RMMA [42]. Thus, this study is in agreement with our results. However, further studies in a larger group are needed to confirm these new risk factors for SB.

The high prevalence of OSA in our study may suggest a secondary form of SB. Interestingly, OSA is also associated with overactivity of the sympathetic system, and prevalence of OSA in the study group was found to be increased in this study. However, we have not observed an association between BEI and AHI; thus, we can say that OSA does not affect the severity of SB. One of the hypotheses concerning SB and OSA is that SB activity is protective against OSA by protruding the mandible and restoring airway patency [43]. Brunelli et al. showed that partial masticatory movements, as in the submaximal opening of mouth by a spring device, causes prolonged reduction of BP and heart rate [44]. Thus, theoretically, SB may also be the element of feedback mechanisms in homeostasis, which prevents excessive surge of BP and tachycardia, usually preceding bruxism episode.

The mean age in both groups was found to be similar; thus, there was no impact of age on the prevalence of SB. The sleepiness assessed using ESS was also similar in both groups. It was found previously that sleepiness as measured by ESS is increased in SB; however, the association of sleepiness with OSA was not assessed in that study [45]. Recently, we have shown that hypertension with OSA does not show increased ESS compared to normotensive subjects [46]; this result agrees with result of this study. It is probable that excessive daytime sleepiness is related not only to the AHI but also to central autonomic interactions affecting baroreflex sensitivity [47,48]. It is worth noting that we used an objective method for diagnosing respiratory disturbance and bruxism. The score in the STOP-BANG questionnaire was found to be increased in the study group, which is predictable because one of the study objectives is related to hypertension. Moreover, the high prevalence of OSA in patients with hypertension also explains the increased STOP-BANG score in the study group of this study.

The sympathetic nervous system plays an important role in the pathogenesis of hypertension. Autonomic cardiovascular control is impaired in hypertension, leading to a reduction in the parasympathetic and an increase in the sympathetic tone [49]. It has been shown that SB is also associated with an increased sympathetic tone. Thus, sympathetic overactivity plays a crucial role in the pathogenesis of both hypertension and SB. Interestingly, patients with SB have an overall elevated sympathetic tone during waking [50]. Thus, similar pathogenesis may contribute of the occurrence of both diseases.

All participants with hypertension were on antihypertensive drugs. There are no data on the influence of antihypertensive medications on the prevalence or severity of bruxism. Beta-adrenoceptor antagonists are used widely to reduce sympathetic tone; thus, these drugs may lead to a decrease in bruxism severity. In addition, spironolactone may decrease bruxism severity as a result of inhibition of the renin–angiotensin–aldosterone system and OSA reduction. However, that aspect was not investigated in this study.

This study has a few limitations. First is the lack of an explanation of the mechanism of increased severity of SB in hypertension. We have not used polysomnography, which is a gold standard for the diagnosis of SB. However, the device we used in this study has been validated for the diagnosis of OSA, and its diagnostic accuracy can be increased by the addition of audio and masseter EMG signals. Another limitation is the absence of ambulatory BP monitoring values for participants. The lack of nocturnal BP measurement and an objectively confirmed diagnosis of hypertension is the most serious limitation of this study; however, the diagnosis of hypertension in the study group was based on an anamnesis which is consistent with the ESH/ESC Practice Guidelines for the Management of Arterial Hypertension [25].

## 5. Conclusions

According to the results of this study, severity but not prevalence of SB was found to be increased in patients with hypertension. Hypertension, higher BMI, lower values of mean SpO_2_, and higher percentages of SpO_2_ < 90% constitute independent risk factors for increased BEI. The results of the study suggest the need for special oral care in patients with hypertension, patients with higher BMI, lower values of mean SpO_2_ and a higher percentage of SpO_2_ < 90%. Further research is highly warranted regarding the pathophysiology of bruxism in arterial hypertension.

## Figures and Tables

**Table 1:** Demographic characteristics of hypertensives (HT) and normotensives (NT).

Parameter	HT (*n* = 35)	NT (*n* = 35)	*P*
Age (years)	57.74 ±7.92	56.94 ± 7.71	0.67
**Body mass (kg)**	**87.56 ± 18.58**	**74.86 ± 15.60**	**0.003**
Height (cm)	173.40 ± 8.15	169.71 ± 10.00	0.10
**BMI (kg/m^2^)**	**28.95 ± 4.63**	**25.88 ± 4.59**	**0.007**
Men (%)	31.4	48.6	0.09
Women (%)	68.6	51.4	0.09
Smoking (%)	29.4	13.3	0.12
Diabetes (%)	17.1	3.0	0.06
CAD (%)	12.1	2.9	0.14
Myocardial infarction (%)	5.7	6.1	0.95
Stroke (%)	0.0	6.1	0.14
ESS	7.71 ± 3.91	7.31 ± 4.40	0.69
**SBS**	**4.94 ± 1.83**	**3.14 ± 1.72**	**0.001**

HT, hypertensives; NT, normotensives; BMI, body mass index (kg/m^2^); CAD, coronary artery disease; ESS, Epworth scale score; SBS, STOP-Bang score; statistically significant differences are marked in a bold font (*p* < 0.05).

**Table 2:** The respiratory and bruxism indices in hypertensives (HT) and normotensives (NT).

Parameter	HT (*n* = 35)	NT (*n* = 35)	*P*
**BEI**	**3.40 ± 3.25**	**2.35 ± 2.29**	**0.04**
**Phasic**	**1.09 ± 1.42**	**0.54 ± 0.76**	**0.04**
Tonic	1.09 ± 1.31	1.01 ± 1.16	0.79
Mixed	1.23 ± 1.49	0.76 ± 1.04	0.13
**AHI**	**19.30 ± 17.59**	**12.39 ± 10.86**	**0.04**
**ODI**	**20.09 ± 17.33**	**12.33 ± 10.50**	**0.03**
**snore (%)**	**16,97 ± 16.31**	**8.73 ± 14.85**	**0.03**
OA	6.35 ± 9.78	3.21 ± 3.79	0.08
MA	0.35 ± 0.98	0.12 ± 0.25	0.20
CA	0.85 ± 1.78	1.09 ± 1.61	0.56
Hypopnea	11.77 ± 9.34	7.95 ± 7.25	0.06
Cheyne-Stokes (%)	0.01 ± 0.08	0.47 ± 2.60	0.31
**Mean SpO_2_**	**92.52 ± 1.75**	**93.69 ± 1.62**	**0.001**
Min SatO_2_	83.03 ± 5.51	85.11 ± 5.22	0.11
**SpO_2_ < 90% (%)**	**10.72 ± 19.36**	**3.47 ± 8.71**	**0.04**
Mean oxygen desaturation	4.45 ± 1.55	4.29 ± 1.35	0.64
Mean heart rate	62.86 ± 8.54	63.07 ± 7.17	0.91
Max heart rate	91.91 ± 16.43	98.26 ± 17.71	0.12
Min heart rate	48.09 ± 8.44	49.49 ± 6.78	0.46

HT, hypertensives; NT, normotensives; BEI, bruxism episode index; AHI, apnea–hypoapnea index; OA, obstructive apnea; MA, mixed apnea; CA, central apnea; ODI, oxygen desaturation index; SpO_2_ < 90% (%), time with oxygen saturation < 90% (% of total bed time); statistically significant differences are marked in a bold font (*p* < 0.05).

**Table 3:** Results of estimation for the final model obtained on multivariate regression analysis.

Model for BEI					
	Intercept	HT	BMI	Mean SpO_2_	SpO_2_ < 90% (%)
Regression coefficient	57.22	2.05	0.12	−0.62	0.05
SEM of Rc	22.41	0.25	0.04	0.13	0.01
*p*-value	0.04	0.02	0.02	0.05	0.02
*p*	0.009				
SEM of model	1.02				

HT, hypertensives; BEI, bruxism episode index; BMI, body mass index; Rc, regression coefficient; SpO_2_ < 90% (%), time with oxygen saturation <9 0% (% of total bed time); SEM, standard error of mean.
